# Gender differences in social networks and physical and mental health: are social relationships more health protective in women than in men?

**DOI:** 10.3389/fpsyg.2023.1216032

**Published:** 2023-12-27

**Authors:** Huiyoung Shin, Chaerim Park

**Affiliations:** Department of Psychology, Jeonbuk National University, Jeonju, Republic of Korea

**Keywords:** social relationships, social networks, physical health, mental health, gender

## Abstract

**Background:**

Individuals’ relationships are characterized by multidimensional aspects and the unique make-up of different features is more or less supportive of physical and mental health. The current study derived social network types based on an extended set of indicators reflecting the structure, function, and quality of relationships, then examined their associations with diverse physical and mental health outcomes separately for men and women.

**Methods:**

Using samples of 620 South Korean adults (*M*_age_ = 53.52), Latent Profile Analysis (LPA) was used to uncover distinct social network types, and multiple regression analyses were conducted to examine the link between network types and health outcomes.

**Results:**

LPA analysis derived four network types: *diversified, family-(un)supported, friend- based*, and *restricted*. The prevalence and configuration of network types differed between men and women: the *family-unsupported* type was more prevalent in women than in men whereas the *restricted* type was more prevalent in men than in women. An individual’s network type membership was significantly associated with one’s physical and mental health and the positive effects of an optimal network type and the negative effects of a non-optimal network type on mental health were much greater for women than they were for men.

**Discussion:**

The findings suggest that women benefit more from supportive networks but that they are also more vulnerable to a lack of supportive (or the presence of conflict-filled) relationships, and highlight that having diversified and greater quality relationships, and avoiding conflicts are critical for women to have enhanced health.

## Introduction

Individuals’ social networks provide them with a key resource over the adult life span as a form of social capital that can influence exchanges of social support (Antonucci et al., 2010). Individuals’ social networks vary in terms of the size and composition of network members as well as in terms of contact frequency with others. These structural aspects of social networks reflect an indication of social integration as well as the available support and resources in times of challenges (Smith and Christakis, 2008). In general, a large and diffuse social network is considered to be more helpful for alleviating difficult problems than a small and family-based social network (Litwin and Shiovitz-Ezra, 2011; Windsor et al., 2016). However, an individual’s social network is multidimensional in nature and characterized by different aspects of the structure, function, and quality of social relationships (Antonucci et al., 2010). Moreover, the features of interpersonal relationships vary by gender and depend on other forms of social capital such as marital status, education, and occupation (Ajrouch et al., 2005). Hence, the presence of social resources could differ by the nature of individuals’ social networks and their interdependent relationships with others.

In this study, we examine how multifaceted features of individuals’ social relationships characterize their distinct social network types, and how these heterotypic network types are related to physical and mental health among both men and women. Specifically, we examine how social resources–as indicated by social network structure, function, and quality–differ by gender and have varying implications on their physical and mental health. This study accordingly assessed multiple indicators of social networks in a sample of South Korean men and women. In deriving distinct network types, a person-oriented latent profile analysis was used to consider interpersonal relationships in a naturally interdependent and aggregated state. After deriving network types, we investigated their links with different aspects of physical and mental health outcomes. Considering an individual’s social networks as a multidimensional construct and investigating their link to multiple aspects of health in this way is expected to illuminate the nuanced associations between social relationships and health. Moreover, by identifying network types separately for men and women, this study could provide a useful lens for understanding the different constellations of interpersonal relationship attributes that characterize men’s and women’s social networks, and describe the effects of network type on health outcomes.

### Multifaceted social networks and health

Individuals’ social networks are often complex, interdependent with multiple social relationships, and characterized by different aspects of structure, function, and quality. As discussed above, the structural aspect of social networks refers to the size and composition of networks, frequency of contact with others, and participation in social activities, which indicate the level of social integration (Smith and Christakis, 2008). Studies focusing on the impact of network structure on health have consistently demonstrated that a high level of social integration has protective benefits for physical and mental health outcomes such as inflammation (Uchino et al., 2018), loneliness (Litwin and Shiovitz-Ezra, 2011), depressive symptoms (García-Peña et al., 2013), life satisfaction, and psychological well-being (Amati et al., 2018).

Meanwhile, the functional aspect of social networks refers to the features or types of social interactions, such as receiving advice and guidance, providing material aid, and expressing comfort and caring (Uchino, 2004). Studies focusing on network functions have also considered the extent of perceived or actual support (Nguyen, 2021) as well as the source of support such as spouse, family, and friends (Ali et al., 2022). Research investigating the impact of network functions on health has shown evidence indicating that both perceived and received support are positively linked to health benefits and that different sources of support are associated with physical and mental health to varying degrees (Windsor et al., 2014; Shin and Gyeong, 2023).

In addition to the network structure and function, the quality of social networks can also play a critical role in individuals’ health outcomes (Uchino, 2006). There is both theoretical and empirical evidence substantiating the claim that social support is not universally beneficial, as the satisfaction and quality associated with such support matters substantially (Holt-Lunstad et al., 2008). Accumulating empirical evidence indicates that perceived quality and satisfaction about relationships have greater impacts on health than the size or composition of social networks (Gallo et al., 2003; Grewen et al., 2005; Shin and Gyeong, 2022).

Despite the empirical support for the importance of considering comprehensive dimensions of social networks in characterizing individuals’ social networks, most prior research on network types has focused on one or two components of social networks (Li and Zhang, 2015; Fang et al., 2020; Ali et al., 2022; Torres et al., 2022; see Fiori et al., 2007, 2008; Nguyen, 2021 for exceptions). Further, in identifying network types, researchers have rarely considered social conflict in tandem with social support despite the fact that conflict and tension can have more potent effects on health than social support (Chen and Feeley, 2014; Shin and Gyeong, 2023). Because individuals maintain different relationships that vary in terms of satisfaction and quality, and because people often experience conflict as well as support within certain relationships (Holt-Lunstad and Uchino, 2019), it can be expected that individuals with the similar structural or functional features of network types can still experience different levels of satisfaction or the levels of support or conflict (Fiori et al., 2007). Such differences in the qualitative aspects or co-occurrence of support and tensions can drive the impact of network types on health outcomes.

### Gender differences in the nature of social networks and health

Research has generally identified four distinct patterns of social networks: a large and diffuse network type characterized by diversified relationships, a relatively large network type characterized by friend-based relationships, a small and narrow network type characterized by family-based relationships, and a restricted and attenuated network type characterized by very few relationships (Fiori et al., 2006; Nguyen, 2021). Depending on the level of diversity and support, researchers have also distinguished sub-clusters within these four network types, including moderately diverse and diverse network types (Windsor et al., 2016) or friend focused-supported and friend focused-unsupported network types (Fiori et al., 2007).

Regardless of the number of network types, distinct network types have been shown to be associated with individuals’ physical and mental health outcomes. Those who were in the diversified network types characterized by interpersonally connected relationships generally reported better health and higher health promoting behaviors along with lower psychological distress, anxiety, depression, and morbidity than those who were in the restricted and attenuated network types (Litwin and Shiovitz-Ezra, 2011; Fang et al., 2020; Wilson et al., 2021). However, scant attention has been paid to possible gender-related differences in the features of network types and their differential link to health outcomes between men and women.

Research has noted the existence of gender differences in social network features, and these differences extend to the structure and function of support networks. In general, women tend to have larger as well as more diffused social networks than men, who tend to have smaller, less intensive, and more limited social networks (Milner et al., 2016; Ang, 2019). Social networks are also assumed to serve different functions for men and women: Women’s social networks are more variable and serve more diverse functions than men’s (Finkel et al., 2018). It has been reported that women generally have more sources for confidant relationships; that is, compared to men, women provide and receive more emotional and health-related support from multiple social ties such as family and friends during times of stress (Liebler and Sandefur, 2002). By contrast, men’s social networks are less affective and intensive than women’s, and men often report that their spouses are their only confidants (Dykstra and de Jong Gierveld, 2004).

Empirical evidence has further indicated that social relationships have different effects by gender, such as the findings that women draw more satisfaction from large and close social networks but that they are also more intensely affected by social events than men (Leach et al., 2008). In general, women are often socialized to be providers of support to multiple network members, and they devote more time and energy to cultivating close interpersonal relationships (Finkel et al., 2018; Ang, 2019). Hence, women can benefit more from the advantages of having access to supportive relationships than men. However, women can also feel increased stress from the additional role they are expected to play as support providers and their involvement in the lives of others (Dalgard et al., 2006). Women can also suffer more when they experience social conflict with close others or experience a lack of support compared to men due to high expectations and devotion toward social relationships (Davis and Greenstein, 2009).

Previous studies have found paradoxical patterns such as those described above, particularly in the context of marital relationships. For instance, research has shown that women feel more responsible toward their spouses than men, which causes women to feel more burdened and overloaded (Neff and Karney, 2005). Greater involvement and feelings of responsibility in family matters and interpersonal problems within the family have been associated with higher depression in women than in men (Orth-Gomér and Leineweber, 2005). Marital relationships lower mortality risks for men but not for women, and the physical health of married men is better than those of their spouses because men benefit more from positive health behaviors that are learned with marriage and their spouses’ efforts to improve health (Donato et al., 2018). By contrast, women often experience everyday social strain in marriage, which leads to greater physiological effects that undermine the health benefits of marriage (Liu and Waite, 2014).

Collectively, the results described above suggest that interpersonal relationships seem to provide women with greater opportunities for more support, which is a protective factor for physical and mental health, but that such relationships are also coupled with increased demands, a greater chance of stresses, and depletion of resources. This suggests that the positive effects of the structural features of social networks could be negated by the burdened roles and demands within those social relationships. Therefore, there is a need for a simultaneous investigation of the structure, function, and quality of diverse relationships (e.g., spouses, family, and friends) as prior research has suggested that both quantitative and qualitative aspects of interpersonal relationships are important, and that men and women have different expectations, experiences, and evaluations of their social relationships (Cornwell, 2011).

Therefore, in the current study, we considered multifaceted aspects of men’s and women’s social networks in both marital and nonmarital forms of interpersonal relationships. By uncovering network types for men and women separately, the results of this study could elucidate which different configurations of structural, functional, and qualitative aspects of marital and nonmarital relationships characterize the nature of gender-specific network types and describe their differential link to diverse physical and mental health outcomes.

### The present study

The overall aim of this study was to uncover distinct network types of men and women and examine their differential associations with physical and mental health. Based on previous evidence (Fiori et al., 2006, 2007; Litwin and Shiovitz-Ezra, 2011), we hypothesized that diversified, family-based, friend-based, and restricted patterns of network types would emerge in the present sample. Moreover, we hypothesized that individuals in network types characterized by diversified relationships and higher relationship quality would exhibit better physical and mental health than individuals in network types characterized by restricted relationships and lower relationship quality. Lastly, we hypothesized that the positive effect of optimal network types and the negative effect of non-optimal network types on health would be greater for women than they would be for men.

## Methods

### Procedures and participants

After receiving approval from the university’s Institution Review Board, we recruited adult participants from an online sampling system that features a panel of 1,663,404 South Korean adults. Using the large sampling pool of respondents living in South Korea, it uses census data to invite, screen, and stratify participants by age and gender. The invitation for recruitment of the national sample was distributed to those who qualified to fill one of 10 subgroups defined by five strata for age (20–29, 30–39, 40–49, 50–59, and 60–69 years) and two strata for gender (men and women) between January 29 and February 5, 2021. Demographic variables such as marital status, retirement status, education, and income were collected but they were not used for stratification. Informed consent was obtained from all participants before they participated in the survey. Participants completed a survey that took about 30 min and they were provided gift certificates upon survey completion. The original sample comprised 1,033 adults (50.1% females) aged between 20 and 69 years. Because individuals aged 40 or over generally tend to be married and have relatively stable relationship patterns, we focused on middle-aged and older adults. The final sample comprised 620 adults (49.19% female; *M*_age_ = 53.52; 40–49 years, *n* = 204; 50–59 years, *n* = 209; 60–69 years, *n* = 207). There were no missing data, and all participants responded to items measuring social network characteristics and health.

### Measures

We used information on the social network characteristics of structure, function, and quality to identify network types, and we set positive and negative physical and mental health as outcomes. Individuals’ demographic information was included as covariates. We used the translated and validated version of the original measures. All of the measures were validated in equivalence in meaning and psychometric properties between the English and Korean versions.

#### Social network characteristics

To assess the *structural* aspect of social networks, we used the Berkman-Syme Social Network Index (Berkman and Syme, 1979). It is a validated measure that assesses an individual’s social integration, including the size of their social network, their marital status, the number of children they have, their contact frequency with their family and friends, and the number of social activities in which they are engaged. We assessed the size and contact frequency with family and friends in terms of the prior 4 weeks. We also included the total number of children and the social activities in which participants were engaged.

To assess the *functional* aspect of social networks, we measured perceived and received support, and conflict. To assess the levels of perceived support, we used the Perceived Social Support Scale (Zimet et al., 1988), which is a 12-item measure that assesses perceived support from family, friends, and close others. A sample item is “I get the emotional help and support I need from my family.” Each item was scored from 1 (not at all true) to 5 (very true). The average score was calculated for each subscale, with higher scores indicating greater perceived support. The Cronbach’s αs for perceived support from family, friends, and close others were 0.91, 0.93, and 0.91, respectively. To assess the levels of received support and conflict, we used the Positive and Negative Social Support Scale (Smith et al., 2017). It is a 24-item measure that assesses support and conflict from one’s spouse, friends, children, and siblings. Sample items are “How much do they really understand the way you feel about things?” for support and “How much do they get on your nerves?” for conflict. Each item was scored from 1 (not at all true) to 5 (very true). The average score was calculated for each subscale, with higher scores indicating greater support and conflict. For the subscales for spouse, friend, child, and sibling, the respective Cronbach’s αs were 0.86, 0.83, 0.82 and 0.88 for support and 0.83, 0.86, 0.83, and 0.90 for conflict.

To assess the *qualitative* aspect of social networks, we measured marital quality and friendship quality. Marital quality was assessed using the Quality Marriage Index (Norton, 1983), which consists of six items measuring the global quality and satisfaction of one’s spousal relationship. A sample item is “I really feel like part of a team with my partner.” Each Item was scored from 1(not at all true) to 5 (very true), and the average score was calculated with higher scores indicating greater marital quality. The Cronbach’s α of this scale in this study was 0.95. Friendship quality was measured using an adapted version of the Friendship Quality Questionnaire (Rose, 2002). The original measure consists of 19 items, and we used 12 items that are applicable to adults that assess positive and negative relationship qualities with friends. A sample item is “I am satisfied with my relationship with my friend.” Each item was scored from 1(not at all true) to 5 (very true), and the average score was calculated with higher scores indicating greater friendship quality. The Cronbach’s α of this scale in this study was 0.90.

#### Health outcomes

We assessed six indicators of health outcomes including physical health, loneliness, depressive symptoms, happiness, life satisfaction, and psychological well-being. *Physical health* was assessed using the Perceived Health Status Scale (Speake et al., 1989), which is a 3-item measure that reflects an individual’s assessment and evaluation of one’s general health. Participants rated the extent to which they agreed with each statement using a 5-point scale (1 = very poor to 5 = very good). A sample statement includes “Generally speaking, would you describe your present health as….” The average score was calculated with higher scores indicating greater health. The Cronbach’s α of this scale in this study was 0.88.

*Loneliness* was assessed using the UCLA Loneliness Scale (Russell et al., 1980), which is a 20-item measure for assessing subjective feelings of loneliness and social isolation. Participants rated the extent to which they agreed with each statement using a 5-point scale (1 = *not at all true* to 5 = *very true*). A sample statement includes “I am unhappy being so withdrawn.” The average score was calculated, with higher scores indicating higher levels of loneliness. The Cronbach’s α of this scale in this study was 0.94. *Depressive symptoms* were assessed using the CES-D Scale (Radloff, 1977). It is a 20-item measure for assessing depressive symptoms experienced over the prior week. A sample statement includes “I felt I could not shake off the blues.” Each item was scored from 0 (*rarely*) to 3 (*most or the time*), and scores were summed to create a scale that ranged from 0 to 60, with higher scores indicating higher levels of depressive symptoms. The Cronbach’s α of this scale in this study was 0.94.

*Happiness* was assessed using the Oxford Happiness Questionnaire developed by Hills and Argyle (2002), which is a 29-item measure to assess levels of happiness as indicated by positive mood. Participants reported to what extent they felt the way explained in each statement using a 5-point scale (1 = *not at all true* to 5 = *very true*). A sample item is “I often experience joy and elation.” The average score was calculated with higher scores indicating greater happiness. Cronbach’s α of this scale in this study was 0.93. *Life satisfaction* was assessed with the Satisfaction with Life Scale (Diener et al., 1985), which is a 5-item measure for assessing global cognitive judgments of one’s life satisfaction. Participants rated the extent to which they agreed with each statement using a 5-point scale (1 *= not at all true* to 5 *= very true*). A sample item is “In most ways my life is close to my ideal.” The average score was calculated, with higher scores indicating greater life satisfaction. The Cronbach’s α of this scale in this study was 0.90.

*Psychological well-being* was assessed with the shortened version of the Psychological Well-being Scale developed by Ryff (1989), which is a 54-item measure comprising six sections to assess levels of autonomy, environmental mastery, personal growth, positive relations with others, purpose in life, and self-acceptance. Participants rated the extent to which they agreed with each statement using a 5-point scale (1 = *not at all true* to 5 = *very true*). A sample item is “In general, I feel confident and positive about myself.” The average score was calculated with higher scores indicating greater psychological well-being. Cronbach’s α in this study was 0.94.

#### Demographic information

The collected demographic information included age, gender, marital status, retirement status, education, and income. Age in years was used as a continuous variable. Gender, marital status, and retirement status were all dichotomized (1 = female; 1 = married; 1 = retired). Education was classified from 1 (≤elementary school) to 5 (graduate school). Income was classified from 1 (≤$10,000) to 5 (5 ≥ $40,000).

### Analytic strategy

All statistical analyses were conducted with SPSS 25.0 and Mplus 8.6. We used SPSS 25.0 for descriptive statistics, independent *t* test, and regression analyses. We used Mplus 8.6 to conduct Latent Profile Analyses (LPA). LPA is a person-oriented analytic method that derives latent profiles into which individuals with similar characteristics can be assigned (Muthén and Muthén, 2000). Identified profiles can be incorporated into the LPA model to build a mixture model to examine the link between profiles and distal outcomes (BCH method; Asparouhov and Muthén, 2021). The LPA analytic models were estimated using full information maximum likelihood estimation (FIML), which treats missing data (e.g., marital quality, number of children) under missing (not) at random assumptions (Asparouhov and Muthén, 2021), allowing derivation of all 620 adults into network types.

Using LPA, we first uncovered network types based on structural, functional, and qualitative aspects of social networks with the total sample. Then we examined whether gender moderate the association between derived network types and health outcomes by running regression models. Results indicated that gender significantly moderated the link between network types and health (e.g., β =0.32; *p* < 0.01 for loneliness). Thus, we proceeded to run LPA using splitted data by gender.

A series of models with profiles from two to five were estimated, separately for men and women, and these models were compared to determine the final solution for the data. After estimating models, we compared multiple fit indices across different profiles based on Akaike Information Criterion (AIC), Bayesian Information Criterion (BIC), sample-size adjusted BIC (SABIC), entropy, and Lo–Mendell–Rubin adjusted likelihood ratio test (LMR-LRT; Collins and Lanza, 2009). We evaluated profile solutions based on the suggestions that the profiles with the lowest AIC, BIC, and SABIC provided the best-fit, and the significant test of LMR-LRT indicated that the *k* + 1 profile was better to the *k*-profile solution. Also, we take into account the entropy values (i.e., the higher the entropy value, the more accurate the profile classification). More importantly, we followed the suggestion that if the additional profile provides a qualitatively differentiated profile that is consistent with the theoretical or empirical evidence to the prior profiles, the new profile should be kept. In contrast, if the additional profile adds only minor differences to the prior profiles, the new profile should not be kept to provide the parsimonious model (Jung and Wickrama, 2008; Marsh et al., 2009; Spurk et al., 2020).

After we identified network types separately for men and women, we investigated if health outcomes in one network type were significantly different from those in other network types using the BCH method (Asparouhov and Muthén, 2021). Then, we conducted multiple regression analyses to examine the link between distinct network types and health outcomes, while controlling for demographic indicators.

## Results

### Gender differences in the nature of social networks and health

To examine gender differences in social network characteristics as well as physical and mental health outcomes, we conducted independent *t* tests and calculated effect-size using Cohen’s *d* for all comparisons. Cohen’s *d* values of 0.2, 0.5, and 0.8 are indicative of small, medium, and large effect sizes, respectively. The results indicated that women reported higher contact frequency with family (*t* = −2.96, *p* < 0.001, *d* = 0.24) and friends (*t* = −2.24, *p* < 0.05, *d* = 0.18), along with higher received support from children (*t* = −5.39, *p* < 0.001, *d* = 0.48), while men reported higher received support from spouse (*t* = 2.54, *p* < 0.05, *d* = 0.23) and higher levels of friend conflict (*t* = 3.79, *p* < 0.001, *d* = 0.31), along with higher levels of relationship satisfaction with their spouses (*t* = 5.09, *p* < 0.001, *d* = 0.47) and friends (*t* = 3.10, *p* < 0.01, *d* = 0.19). In terms of physical and mental health, a significant gender difference was found in life satisfaction: men reported greater life satisfaction than women (*t* = 3.49, *p* < 0.01, *d* = 0.28).

### Latent profiles of network types in men and women

Network types were identified separately for men and women. We provided the fit indices and profile distributions for different models in Table 1. For men, results indicated that the three- to five- profile models appropriately fit the data. They indicated high entropy values (0.91–0.92), showing high levels of profile classification accuracy. Although the AIC, BIC, and SABIC values suggest that the five-profile model showed a better fit, the small size of the five-profile solution (4.76% of the sample) provides statistical justification for the four-profile model. For women, results indicated that the two- to five- profile models appropriately fit the data. They indicated high entropy values (0.88–0.93), showing high levels of profile classification accuracy. Although the significant test of LMR-LRT showed that the two-profile solution was better to the three- or four-profile solution, the AIC, BIC, and SABIC values suggest that the four- or five-profile model showed a better fit. While the five-profile solution marginally improved the overall fit, this model produced one nearly identical and less differentiated profile that contained only 6 to 15 cases. In contrast, the four-profile solution provided a substantively meaningful profile that aligns well with existing literature. Thus, based on the interpretability and meaningfulness of profiles, we selected the four-profile solutions as the final model for men and women. The entropy of the final model was 0.91 and 0.88 for men and women, respectively, indicating that 91 and 88% of the men’s and women’s sample were correctly classified.

**Table 1:** Fit indices and profile distributions for different latent profile solutions.

		Fit indices					Profile proportion (%)	
		AIC	BIC	SABIC	Entropy	LMR-LRT	Max	Min
Men (*n* = 315)	2 Profiles	15057.68	15286.59	15093.11	0.87	*p* = 0.15	57.46	42.54
	3 Profiles	14559.39	14867.10	14607.02	0.92	*p* < 0.01	53.33	19.68
	4 Profiles	14336.48	14722.99	14396.31	0.91	*p* < 0.05	40.32	15.56
	5 Profiles	14144.89	14610.21	14216.92	0.91	*p* = 0.30	38.41	4.76
Women (*n* = 305)	2 Profiles	15023.37	15250.31	15056.85	0.93	*p* < 0.001	71.80	28.20
	3 Profiles	14801.98	15107.04	14846.00	0.91	*p* = 0.22	56.07	17.38
	4 Profiles	14631.04	15014.23	14687.56	0.88	*p* = 0.75	41.97	16.39
	5 Profiles	14497.93	14959.25	14565.98	0.91	*p* = 0.45	31.80	14.10

Note: AIC, Akaike Information Criteria; BIC, Bayesian Information Criteria; SABIC, sample adjusted BIC; LMR-LRT, Lo-Mendell-Rubin likelihood ratio test.

**Figure 1:**
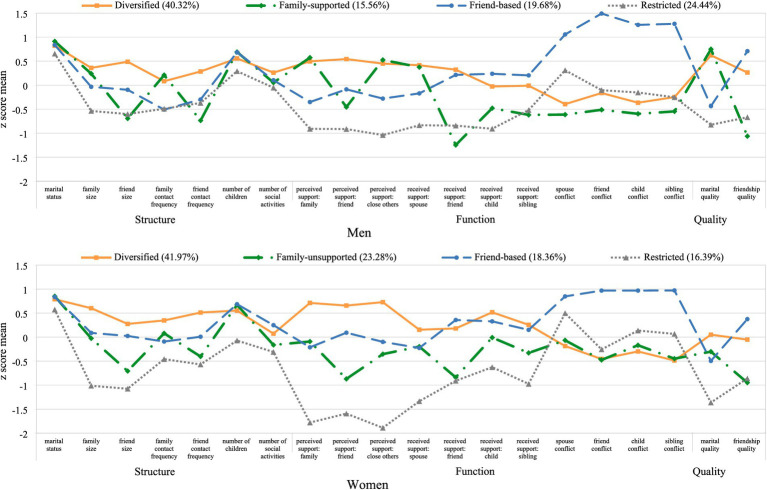
Identified social network types in men and women. Note: For marital status, we used the original score of 0 (*not married*) and 1 (*married*).

As provided in Figure 1, four network types emerged for men and women, respectively: *diversified*, *family-(un)supported*, *friend-based*, and *restricted*, which correspond to the network types that have generally found in the literature. However, between men and women, the proportion ( $\chi^{2}$ = 9.92, *p* < 0.05) and composition of network types differed, specifically for *family-(un)supported* and *restricted* network types; we discuss the both common and distinctive features of the gender-specific descriptions of each network type below.

The *diversified* network type comprised individuals with the highest network size and contact frequency in terms of family and friends. They reported the highest levels of support and below-average levels of conflict from all relationship sources, as well as above-average levels of marital and friendship quality. The *diversified* network type was the most prevalent type in both men (40.32%) and women (41.97%). Individuals in the *family-(un)supported* network type had an average network size and contact frequency with family but below-average network size and contact frequency with friends. Although men in this type (i.e., *family-supported;* 15.56%) reported higher perceived support from family, spouse, and close others, and the highest marital quality, women in this type (i.e., *family-unsupported;* 23.28%) reported below-average perceived support from family, spouse, and close others, and reported below-average martial quality.

The *friend-based* network type comprised individuals with an average network size and the highest engagement in social activities. Individuals in this type reported above-average received support from children, siblings, and friends, but below average perceived support from spouses, family, and close others. Notably, they described their interpersonal relationships as highly negative; they reported the highest levels of conflicts with spouses, family, and friends. Moreover, although they reported the highest levels of friendship quality, they reported below-average levels of marital quality. The *friend-based* network type was similarly prevalent in men (19.68%) and in women (18.36%). Individuals in the *restricted* network type had comparatively small social networks. They had infrequent contacts with family and friends, reported low levels of perceived and received support, and had above-average spousal conflict as well as the lowest marital quality. Although the *restricted* network type was more prevalent in men (24.44%) than in women (16.39%), women in this type reported substantially lower perceived support from all relationship sources as well as lower marital quality than men in this type.

### Differences in physical and mental health by network type

**Figure 2:**
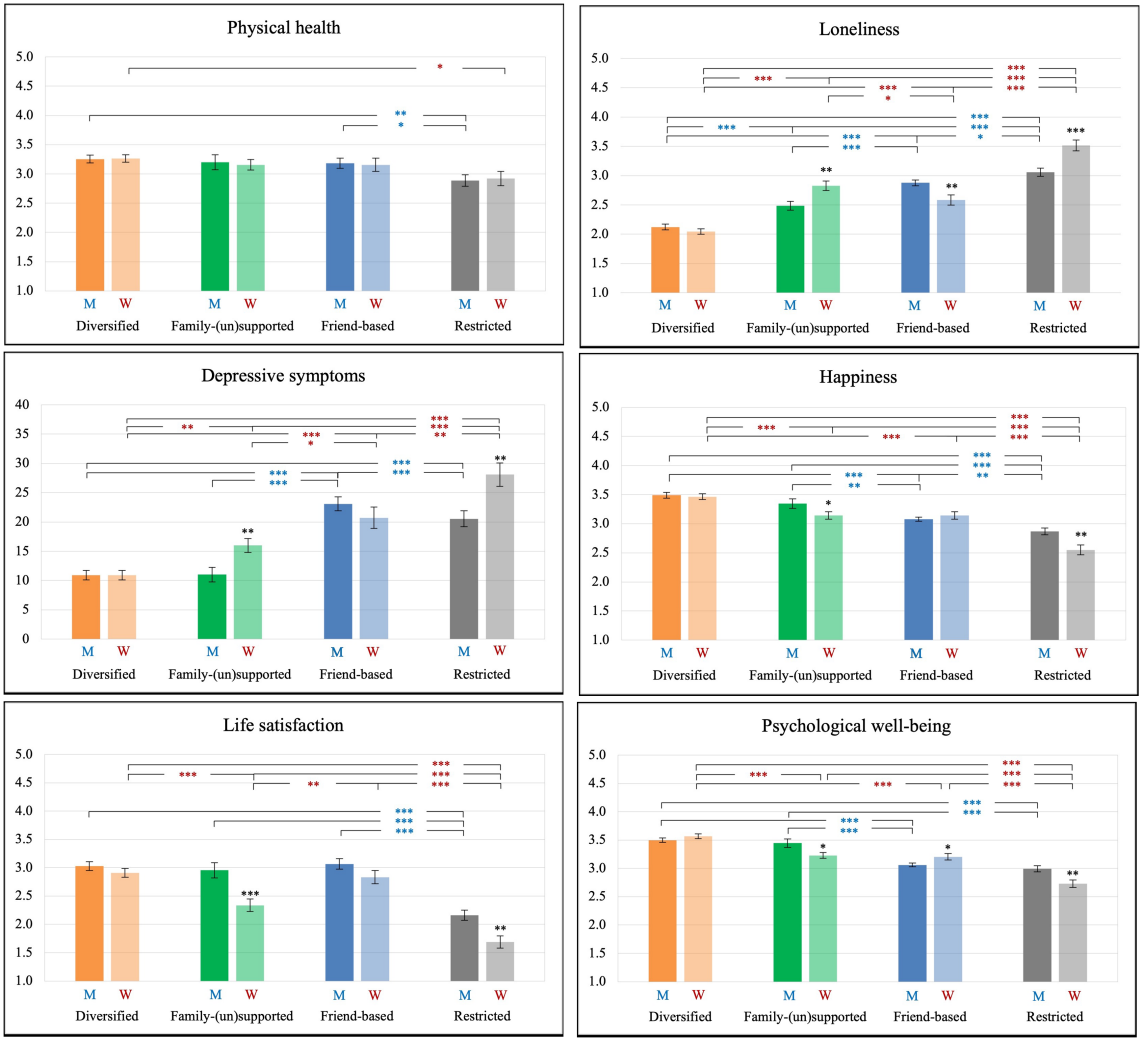
Significant differences in mean levels of physical and mental health outcomes across four network types in men and women. Note: M, ^*^ = men; W, ^*^ = Women; Analyses were conducted with the BCH method; Differences in mean scores of physical and mental health outcomes between network types were significant at ^*^p < 0.05. ^**^p < 0.01. ^***^p < 0.001; Gender differences within each network type were also reported above the bar of women. ^*^p < 0.05. ^**^p < 0.01. ^***^p < 0.001.

As presented in Figure 2, we found significant mean level differences in physical and mental health outcomes across the four network types. In general, for both men and women, individuals in the *diversified* network type had the highest levels of physical health, happiness, and psychological well-being, and the lowest levels of loneliness and depressive symptoms, followed by individuals in the *family-(un)supported*, *friend-based*, and *restricted* network types. The overall pattern was similar between men and women, but there were significant gender differences in the mean levels of mental health outcomes, mostly in the *family-(un)supported* and *restricted* network types; compared to men in the *family-supported* and *restricted* network types, women in the *family-unsupported* and *restricted* network types reported higher loneliness and depressive symptoms as well as lower happiness, life satisfaction, and psychological well-being.

### Associations of network types with physical and mental health

As presented in Tables 2, 3, an individual’s network type membership was found to be significantly associated with their physical and mental health outcomes, after controlling for demographic information (i.e., age, marital status, retirement status, education, and income). Specifically, compared to being in the *restricted* network type (which was a reference group in the regression model), being in the *diversified* and *family-(un)supported* network type was associated with higher levels of happiness, life-satisfaction, and psychological well-being, along with lower levels of loneliness and depressive symptoms. Moreover, compared to being in the *restricted* network type, being in the *friend-based* network type was associated with higher levels of happiness and life-satisfaction. In women only, being in the *friend-based* network type was additionally associated with higher levels of psychological well-being and lower levels of loneliness and depressive symptoms. Although the general pattern of the associations between network types and health outcomes was comparable between men and women, the effect sizes of the network type impact on health were greater in women than they were in men.

**Table 2:** Regression coefficients for predicting physical health, loneliness, and depressive symptoms.

	Physical health		Loneliness		Depressive symptoms	
	$\beta_{\mathrm{men}}$	$\beta_{\mathrm{women}}$	$\beta_{\mathrm{men}}$	$\beta_{\mathrm{women}}$	$\beta_{\mathrm{men}}$	$\beta_{\mathrm{women}}$
**Social network type** ^a^						
Diversified	0.18^*^	0.19^*^	−0.68^***^	−0.91^***^	−0.40^***^	−0.65^***^
Family-(un)supported	0.08	0.12	−0.31^***^	−0.37^***^	−0.28^***^	−0.40^***^
Friend-based	0.10	0.11	−0.10^†^	−0.46^***^	0.09	−0.24^**^
Age	0.03	0.11	−0.09	0.01	−0.18^**^	−0.09
Marital status^b^	0.16^*^	0.02	−0.08	−0.02	−0.10^†^	0.01
Retirement status^c^	−0.05	−0.11	0.11^*^	−0.05	0.07	0.10^†^
Education	0.09	0.13^*^	0.04	−0.03	0.01	−0.06
Income	0.02	0.05	0.01	−0.03	−0.04	−0.10^†^
*R*^2^	0.08	0.05	0.37	0.44	0.27	0.26
*adj R*^2^	0.05	0.03	0.36	0.43	0.25	0.24
*F*	3.16^**^	2.04^*^	22.94^***^	29.34^***^	14.35^***^	12.79^***^
Cohen’s *f*^2^	0.09	0.05	0.59	0.79	0.37	0.35

Note: β = standardized coefficient; *R*^2^ = the proportion of the variance in the dependent variable explained by the independent variables in the model, serving as an indicator of model fit; adj *R*^2^ = Adjusted R^2^ is modification of R^2^ that considers the number of independent variables in the model.

a Restricted network type was specified as the reference group.

b Marital status is coded 0 = not married.

c Retirement status is coded 0 = not retired.

The scale ranges are as follows: age = 40–69; education, 1≤ elementary school, 2 = middle school, 3 = high school, 4 = some college, 5 = graduate school; income, 1≤ $10,000, 2 = $10,000–$20,000, 3 = $20,000–$30,000, 4 = $30,000–$40,000, 5 ≥ $40,000; physical health, 1–5; loneliness, 1–5; depressive symptoms, 0–3. ^†^*p* < 0.10. ^*^p < 0.05. ^**^p < 0.01. ^***^p < 0.001.

**Table 3:** Regression coefficients for predicting happiness, life satisfaction, and psychological well-being.

	Happiness		Life satisfaction		Psychological well-being	
	$\beta_{\mathrm{men}}$	$\beta_{\mathrm{women}}$	$\beta_{\mathrm{men}}$	$\beta_{\mathrm{women}}$	$\beta_{\mathrm{men}}$	$\beta_{\mathrm{women}}$
**Social network type** ^a^						
Diversified	0.52^***^	0.69^***^	0.41^***^	0.60^***^	0.48^***^	0.74^***^
Family-(un)supported	0.28^***^	0.39^***^	0.25^***^	0.28^***^	0.30^***^	0.38^***^
Friend-based	0.13^*^	0.37^***^	0.33^***^	0.45^***^	0.04	0.35^***^
Age	0.10	0.14^*^	−0.07	0.02	0.09	0.08
Marital status^b^	0.10^†^	0.01	0.11^†^	0.05	0.11^†^	−0.02
Retirement status^c^	−0.02	−0.08	0.07	−0.06	−0.02	0.01
Education	0.03	0.18^***^	0.12^*^	0.17^**^	0.06	0.17^**^
Income	0.05	0.11^*^	0.11^*^	0.06	0.02	0.09^†^
*R*^2^	0.24	0.31	0.21	0.26	0.25	0.33
*adj R*^2^	0.22	0.29	0.19	0.24	0.23	0.31
*F*	12.07^***^	16.75^***^	10.41^***^	12.69^***^	12.99^***^	18.23^***^
Cohen’s *f*^2^	0.32	0.45	0.27	0.35	0.33	0.49

Note: β = standardized coefficient; *R*^2^ = the proportion of the variance in the dependent variable explained by the independent variables in the model, serving as an indicator of model fit; adj *R*^2^ = Adjusted R^2^ is modification of R^2^ that considers the number of independent variables in the model.

a Restricted network type was specified as the reference group.

b Marital status is coded 0 = not married.

c Retirement status is coded 0 = not retired.

The scale ranges are as follows: age = 40–69; education, 1≤ elementary school, 2 = middle school, 3 = high school, 4 = some college, 5 = graduate school; income, 1≤ $10,000, 2 = $10,000–$20,000, 3 = $20,000–$30,000, 4 = $30,000–$40,000, 5 ≥ $40,000; happiness, 1–5; life satisfaction, 1–5; psychological well-being, 1–5. ^†^*p* < 0.10. ^*^p < 0.05. ^**^p < 0.01. ^***^p < 0.001.

## Discussion

In this study, we identified distinct network types among men and women that reflect different configurations of structural, functional, and qualitative aspects of relationships. We then examined whether such heterotypic network types were differentially associated with diverse physical and mental health outcomes. By incorporating a person-oriented analysis and considering different aspects of relationship characteristics in identifying network types, we were able to achieve a more nuanced understanding of individuals’ different relationship profiles.

### Latent profiles of network types in men and women

In line with the results of previous research (Litwin and Shiovitz-Ezra, 2011; Wilson et al., 2021) as well as our hypotheses, we uncovered four distinct network types: *diversified*, *family-(un)supported*, *friend-based*, and *restricted*. However, the prevalence and composition of network types differed between men and women. For instance, the *family-unsupported* network type was found to be more prevalent in women than in men, and the nature of relationships were more positive in men’s *family-supported* than in women’s *family-unsupported*. Compared to men in this type, women in this type reported lower perceived support from family and spouses, as well as lower marital quality. These findings are consistent with the idea that women, compared to men, generally provide more support to their family and spouses whereas they receive less support from their spouses and close others (Neff and Karney, 2005; Leach et al., 2008).

We also found some differences by gender in terms of the *restricted* network type; specifically, it was more prevalent in men than it was in women, which is in line with previous evidence showing that the social networks of men are more limited and smaller than those of women (Finkel et al., 2018; Ang, 2019). However, although less women than men belonged to the *restricted* network type, women in this type reported a much smaller network size, substantially lower perceived and received support from family and friends, along with lower marital quality than men in this type. These women could be considered to be individuals who are at risk for health problems given that women are more vulnerable to a lack of supportive networks due to their higher expectations and emphasis toward social relationships and emotional closeness than men (Davis and Greenstein, 2009). This idea is further substantiated by our findings (i.e., women in this type reported higher loneliness and depressive symptoms, as well as lower happiness, life satisfaction and psychological well-being than men in this type).

The *friend-based* network type was similarly prevalent in men and women. Men and women in this type both reported below-average perceived support from family and below-average marital quality. Although both men and women in this type reported above-average conflict with family and friends, men in this type reported relatively higher conflict than women in this type. Given that the *friend-based* network type comprised individuals with the highest social engagement, these findings are consistent with the idea that spread-out social networks can provide not just benefits but also costs (Antonucci et al., 2010; Wan and Antonucci, 2016). That is, extended social networks could impose added obligations on an individual and multiple relationships may serve to exacerbate stresses or conflicts because interpersonal relationships are costly in nature (e.g., energy expenditure) and have possible risks (e.g., negative affect). And, men can experience added stress from the additional relationships and social roles because they draw less satisfaction from large and diverse social networks than women (Ang, 2019).

The *diversified* network type was the most prevalent type in both men and women, and the composition of the network structure and function was also comparable between men and women in this type. Individuals in this type reported frequent contact with family and friends, above-average perceived and received support, and below-average conflict with all relationship sources. In terms of relationship quality among individuals in this type, men reported higher marital quality than women. Collectively, the results of the current study suggested that the *diversified* network type was the most common and optimal network type in both men and women. And, the prevalence and features of network types differed by gender, particularly within the *family-(un)supported* and *restricted* network types; this could drive the impact of network types on men’s and women’s health outcomes, which is further discussed below.

### Associations of network types with physical and mental health

As anticipated, heterotypic network types–which are characterized by different constellations of social relationship features–were differentially associated with diverse physical and mental health outcomes. Individuals in the *diversified* network types reported the highest levels of physical health, happiness, and psychological well-being, as well as the lowest levels of loneliness and depressive symptoms, followed by individuals in the *family-(un)supported*, *friend-based*, and *restricted* network types. These findings could be related to the theory and research emphasizing the importance of structural aspects of social networks (Moen, 2001). Our findings support that *diversified* social networks indeed have beneficial effects in promoting physical and mental health. Because having multiple relationship sources and a broader network structure indicates the availability of more support and resources in times of challenges, individuals in the *diversified* network types could find helpful solutions for difficult problems more easily than those in small or *restricted* network types (Windsor et al., 2016).

For men, individuals in the *family-supported* network type had significantly higher happiness, and psychological well-being and lower loneliness, and depressive symptoms than those in the *friend-based* network type. In contrast, for women, although individuals in the *family-unsupported* network type had similar levels of physical health and happiness as those in the *friend-based* network type, they had significantly higher loneliness but lower depressive symptoms, and life-satisfaction than those in the *friend- friend-based* network type. Such findings should be interpreted in consideration of the fact that the *friend-based* network type was characterized by both high levels of support and conflict with family and friends. Although high engagement in the community and increased interactions with friends can reduce perceived isolation and provide positive emotions (Cohen and Pressman, 2006), extended social interactions across multiple relationships can also induce conflict and tension. Because increased social strain can lead to detrimental effects on health that outweigh the benefits of support, accumulated conflict within enlarged networks is likely to reduce the positive effects of support and lead to increased depressive symptoms and lower life satisfaction.

The overall patterns of the associations between network types and health outcomes were found to be similar between men and women. However, the positive effects of an optimal network type and the negative effects of a non-optimal network type on health outcomes were much greater for women than they were for men. This finding highlights that women benefit more from supportive social networks but also are more vulnerable to a lack of supportive (or the presence of conflict-filled) relationships (Dalgard et al., 2006; Leach et al., 2008). Interestingly, the findings further showed that belonging to the *friend-based* network type was linked to lower loneliness and depressive symptoms as well as higher psychological well-being only for women, and not for men. These findings can be interpreted to indicate that, for women, relationship with friends do have significant effects on their physical and mental health. It is likely that men and women are differentially affected by friend support. Given that women generally have more confidant relationships with friends than men, and that women’s psychological well-being is more closely related to the positive and negative aspects of both marital and friend relationships than that of men (Liebler and Sandefur, 2002).

Among individuals in the *family-(un)supported* network type, women generally reported lower marital quality than men, along with significantly higher loneliness and depressive symptoms than men. These results suggest that women–particularly those who belong to the *family-unsupported* type–have greater involvement and responsibility for taking care of their spouses and family, which can cause them to feel more burdened and thus have more mental health problems, such as depressive symptoms (Orth-Gomér and Leineweber, 2005). With the modernization of South Korea, women in the current society tend to have higher educational status, and have a more active role in the labor market than women in the past (Lee et al., 2008). However, because public support for childcare services has been lacking, women who are socially active should have additional burden of taking care of their child and family in addition to their work responsibilities (Hyun et al., 2002). Thus, although modernization has provided women with more opportunities, women with multiple roles and responsibilities could be more burdened and overloaded.

The existing body of research has demonstrated that women who experience everyday social strain and challenges in marriage can have increased physiological problems that undermine the health benefits of marriage (Liu and Waite, 2014). The current findings further substantiate that perceived quality and satisfaction about relationships can have more critical impacts on women’s health than the size or composition of networks (Grewen et al., 2005; Holt-Lunstad et al., 2008). Given that relationship quality varied by network type and gender, and that those who belonged to network types characterized by high relationship quality reported positive health outcomes, it is likely that the positive health benefits of belonging to an optimal network type mainly stem from the perceived support, emotional closeness, and care from their supportive networks.

The current study has limitations that should be noted. First, this study was based on a cross-sectional research design. Thus, caution should be taken when interpreting the directionality of the research variables. Second, although the current sample is deemed to be representative of South Korean adult population based on age and gender with the use of a stratified probability sampling, it might not be representative in all respects. For instance, the type of individuals who would be willing to participate in a research platform may not be typical of the general population. Future research should replicate our results based on different cohorts using diverse methods of administration (online vs. face-to-face) to allow for the generalization of the link between network types and health.

Despite the limitations, this study makes an important contribution to the literature by uncovering distinct network types of men and women based on multidimensional aspects of social relationships. Substantial evidence points to the robust linkage between relationships and health (Uchino, 2013). A large body of this work considers different aspects of social relationships and highlights the structural or functional components of relationships as having significance for health in adulthood. This study builds on the importance of relationships for health by considering multifaceted social network features and an extended set of health outcomes separately for men and women. Results from this study reveal that the effect of social relationships on health could be contingent upon gender. Many previous studies, either ignored or controlled for gender as a covariate. By examining different associations between heterotypic network types and diverse physical and mental health and important gender-related differences in these associations, we found that men and women may actually perceive and experience social relationships differently, and that such differences become important in shaping their health.

The current findings provide important practical implications. Our profile-based approach identified women who belonged to the *restricted* type; these women reported having attenuated social networks characterized by very few social relationships and experiencing low perceived and received support from family and friends. Those women who may need help but not having close or supportive confidants also reported high levels of loneliness and depressive symptoms. These women could be considered to be individuals who are at risk for developing more serious mental health problems. Based on the results, health practitioners should aim to distinguish these women from others as a means to detect and assist at-risk group of individuals.

Our results further suggest that friends are influential and important social relationships, especially for women. Our findings demonstrate that even though women experience some levels of tension and conflict with friends, their mental health is closely related to the positive and negative aspects of friendships. As people age, they rely on fewer relationship partners for social and emotional needs. Having recognized this, our findings underscore that older adults (especially women) should be encouraged to develop and maintain supportive relationships with friends because they are important social resources to promote positive mental health. Implementation of programs designed to promote social interactions within their existing social networks may reduce the risks of mental health problems, especially for women at old ages.

## Data availability statement

The raw data supporting the conclusions of this article will be made available by the authors, with modest request.

## Ethics statement

The studies involving humans were approved by Jeonbuk National University/IRB. The studies were conducted in accordance with the local legislation and institutional requirements. The participants provided their written informed consent to participate in this study.

## Author contributions

HS conceived of the study, helped analyses and interpretation of the data, and drafted the manuscript. CP did analyses and interpreted the data. All authors contributed to the article and approved the submitted version.
