# Supplementary material for: Gender differences in social networks and physical and mental health: are social relationships more health protective in women than in men?
Source: Front Psychol. 2023 Dec 27;14:1216032. doi: 10.3389/fpsyg.2023.1216032 (PMC10782512; doi:10.3389/fpsyg.2023.1216032)
Supplement: Supplementary file 1 [file Table_1.docx]

Appendix

Figure A.1.

Stratified sampling information

**Online panel data sampling**

(*n*=1,033)

**20s**

(*n*=100)

**30s**

(*n*=100)

**40s**

(*n*=104)

**50s**

(*n*=105)

**60s**

(*n*=106)

**Female**

(*n*=518)

**20s**

(*n*=110)

**30s**

(*n*=103)

**40s**

(*n*=100)

**50s**

(*n*=104)

**60s**

(*n*=101)

**Male**

(*n*=515)

**Online panel of South Korean adults**

(*n=*1,663,404)

Table A.1.

Demographic information of participants

|  | 40s | 50s | 60s | All |
| --- | --- | --- | --- | --- |
|  |  |  |  |  |
| Sample size (*N*) | 204 | 209 | 207 | 620 |
| Age, *mean (SD)* | 43.97 (2.91) | 53.20 (2.70) | 63.27 (2.73) | 53.52 (8.34) |
| Gender |  |  |  |  |
| Male, *N* (*%*) | 104 (33%) | 105 (33%) | 106 (34%) | 315 |
| Female, *N* (*%*) | 100 (33%) | 104 (34%) | 101 (33%) | 305 |
| Education |  |  |  |  |
| < High school, *N* (*%*) | 37 (26%) | 47 (32%) | 61 (42%) | 145 |
| Some college, *N* (*%*) | 149 (37%) | 133 (33%) | 120 (30%) | 402 |
| Graduate school, *N* (*%*) | 18 (25%) | 29 (40%) | 26 (36%) | 73 |
| Income |  |  |  |  |
| < $10,000, *N* (*%*) | 33 (23%) | 56 (40%) | 52 (37%) | 141 |
| $10,000-$20,000, *N* (*%*) | 15 (28%) | 14 (26%) | 25 (46%) | 54 |
| $20,000-$30,000, *N* (*%*) | 24 (30%) | 27 (34%) | 28 (35%) | 79 |
| $30,000-$40,000, *N* (*%*) | 34 (37%) | 21 (23%) | 38 (41%) | 93 |
| > $40,000, *N* (*%*) | 98 (39%) | 91 (36%) | 64 (25%) | 253 |
| Retirement status |  |  |  |  |
| Retired, *N* (*%*) | 11 (7%) | 26 (16%) | 125 (77%) | 162 |
| Not retired, *N* (*%*) | 193 (42%) | 183 (40%) | 82 (18%) | 458 |
| Marital status |  |  |  |  |
| Married, *N* (*%*) | 145 (30%) | 171 (35%) | 172 (35%) | 488 |
| Single, *N* (*%*) | 59 (45%) | 38 (29%) | 35 (27%) | 132 |
| Children |  |  |  |  |
| One or more, *N* (*%*) | 142 (27%) | 183 (35%) | 195 (38%) | 520 |
| None, *N* (*%*) | 62 (62%) | 26 (26%) | 12 (12%) | 100 |
|  |  |  |  |  |

Table A.2.

Group means and proportion for entire social network indicators for four network types in men and women

|  | *Diversified* | |  | *Family-(un)supported* | |  | *Friend-based* | |  | *Restricted* | |
| --- | --- | --- | --- | --- | --- | --- | --- | --- | --- | --- | --- |
|  | Men | Women |  | Men | Women |  | Men | Women |  | Men | Women |
| Structure |  |  |  |  |  |  |  |  |  |  |  |
| 1. Married (proportion) | 0.82 | 0.78 |  | 0.92 | 0.86 |  | 0.84 | 0.84 |  | 0.65 | 0.58 |
| 1. Family size | **3.67** | **3.93** |  | **3.58** | 3.27 |  | 3.31 | 3.42 |  | 2.82 | **2.39** |
| 1. Friend size | **3.35** | **3.13** |  | **2.09** | **2.03** |  | 2.72 | 2.83 |  | **2.14** | **1.63** |
| 1. Family contact frequency | 3.94 | 4.43 |  | 4.22 | 3.89 |  | 3.03 | 3.68 |  | 3.08 | **3.18** |
| 1. Friend contact frequency | 3.39 | 3.74 |  | **1.86** | 2.30 |  | 2.50 | 2.96 |  | 2.36 | **2.08** |
| 1. Number of children | 1.61 | 1.59 |  | 1.73 | 1.76 |  | 1.74 | 1.75 |  | 1.34 | **0.98** |
| 1. Number of social activities | 1.72 | 1.55 |  | 1.51 | 1.37 |  | 1.60 | 1.70 |  | 1.45 | 1.24 |
| Function |  |  |  |  |  |  |  |  |  |  |  |
| 1. Perceived support: family | **4.13** | **4.33** |  | **4.22** | 3.59 |  | 3.34 | 3.48 |  | **2.82** | **2.01** |
| 1. Perceived support: friend | **3.88** | **3.99** |  | 2.91 | **2.50** |  | 3.27 | 3.43 |  | **2.45** | **1.79** |
| 1. Perceived support: close others | **4.00** | **4.27** |  | **4.09** | 3.22 |  | 3.31 | 3.50 |  | **2.58** | **1.78** |
| 1. Received support: spouse | 3.96 | 3.70 |  | 3.94 | 3.43 |  | 3.38 | 3.33 |  | **2.75** | **2.23** |
| 1. Received support: friend | **3.44** | 3.29 |  | **2.07** | **2.43** |  | 3.34 | 3.45 |  | **2.41** | **2.39** |
| 1. Received support: child | 2.82 | 3.32 |  | 2.40 | 2.87 |  | **3.06** | 3.14 |  | **2.01** | **2.26** |
| 1. Received support: sibling | 2.79 | 3.05 |  | **2.17** | 2.47 |  | 3.02 | 2.98 |  | 2.27 | **1.81** |
| 1. Spouse conflict | 1.98 | 2.17 |  | **1.81** | 2.28 |  | **3.33** | **3.16** |  | 2.63 | 2.81 |
| 1. Friend conflict | 1.67 | 1.42 |  | **1.39** | 1.39 |  | **3.09** | **2.65** |  | 1.72 | 1.60 |
| 1. Child conflict | 1.68 | 1.73 |  | **1.46** | 1.82 |  | **3.08** | **2.85** |  | 1.86 | 2.12 |
| 1. Sibling conflict | 1.59 | 1.39 |  | **1.35** | 1.41 |  | **2.94** | **2.67** |  | 1.60 | 1.88 |
| Quality |  |  |  |  |  |  |  |  |  |  |  |
| 1. Marital quality | **4.31** | 3.74 |  | **4.43** | 3.41 |  | **3.23** | 3.16 |  | **2.85** | **2.30** |
| 1. Friendship quality | 2.94 | 2.71 |  | **2.01** | 2.06 |  | **3.24** | 3.01 |  | **2.26** | **2.15** |
|  |  |  |  |  |  |  |  |  |  |  |  |

*Note.* Boldfaced numbers indicate defining peaks of the profile types (specifically, approximately 0.5 or > 0.5 *SD* above or below the sample mean). The scale ranges are as follows: family and friend size, 1–6; family and friend contact frequency, 1–6; number of children, 0–4; number of social activities, 1–6; perceived support, 1–5; received support, 1–5; conflict, 1–5; marital quality, 1–5; friendship quality, 1–5.
